# Addressing COVID-19 in Inmates at Women’s State Prison in Indonesia

**DOI:** 10.3390/tropicalmed11100268

**Published:** 2026-09-27

**Authors:** Al Asyary, Aisyah Rizqi Rahimullah, Budi Haryanto, Harunor Rashid

**Affiliations:** 1 Department of Environmental Health, Faculty of Public Heath, University of Indonesia, 2nd Floor Building C, Campus UI, Depok 16424, Indonesia; bharyanto@ui.ac.id; 2 FED Indonesia Co., Ltd., Jakarta 10210, Indonesia; aisyahrzqii@gmail.com; 3 Steering Council, Mass Gathering Medicine Interest Group, International Society of Travel Medicine (ISTM), Atlanta, GA 30009, USA; harunor.rashid@sydney.edu.au; 4 The Children’s Hospital at Westmead Clinical School, Faculty of Medicine and Health, The University of Sydney, Westmead, NSW 2145, Australia; 5 Sydney Infectious Diseases Institute, The University of Sydney, Westmead, NSW 2145, Australia

**Keywords:** COVID-19, contact history, incarceration, mass gathering, prison

## Abstract

Coronavirus Disease 2019 (COVID-19), caused by Severe Acute Respiratory Syndrome Coronavirus 2 (SARS-CoV-2), is characterized by symptoms such as cough, fever, fatigue, and myalgia. COVID-19 became widespread in Indonesia, with the highest incidence recorded in the Daerah Khusus Ibukota (DKI) Jakarta Province. The movement of individuals into and out of prisons, overcrowding, inadequate sanitation, and limited access to fresh air and physical activities, among other factors, contribute to the high risk of COVID-19 transmission in correctional facilities. Consequently, this study examines the frequency of COVID-19 among prisoners at the Women’s State Prison in Jakarta. Responses from a previously published survey were analyzed. The research employed a cross-sectional study design, surveying 105 prisoners within the facility. The primary data were obtained through completion of a structured questionnaire. Chi-square analysis demonstrated a significant association between a history of contact and COVID-19 infection (*p* < 0.01, odds ratio [OR] = 17.21). In contrast, conventional COVID-19 preventive behaviors (*p* = 0.35, OR = 1.70) and access to facilities and amenities (*p* = 0.77, OR = 1.23) were not significantly associated with COVID-19 infection among inmates. These findings have important policy implications for the prevention and control of epidemics in female prisoners in Indonesia.

## 1. Introduction

In late December 2019, an enigmatic pneumonia outbreak, later identified as coronavirus disease 2019 (COVID-19), was first detected at the Huanan seafood wholesale market in Wuhan, China, before rapidly disseminating worldwide [1]. The World Health Organization (WHO) subsequently declared COVID-19 a global pandemic, whereas the Indonesian government categorized it as a non-natural disaster resulting from a disease outbreak [2,3]. As of 20 January 2023, Indonesia had reported 6,727,609 confirmed cases and 160,772 deaths. Cases had spread to nearly all regions of the country, with the Daerah Khusus Ibukota (DKI) Jakarta Province reporting the highest cumulative number of cases (1,141,024) [4,5].

Prisons are among the highest risk settings for COVID-19 transmission. The likelihood of COVID-19 infection in correctional facilities is influenced by factors such as overcrowding, health literacy, and hygienic and environmental conditions [6]. As reported by the Ministry of Law and Human Rights of the Republic of Indonesia in August 2021, the nation’s correctional facilities were operating at 103% overcapacity [7]. Such overcrowding creates conditions that enable the rapid and widespread transmission of SARS-CoV-2 following the introduction of an index case. Furthermore, limited access to healthcare services and facilities, the continual movement of prison staff, family members, legal staff, and other visitors between prisons and the community, inadequate hand hygiene, and poor compliance with respiratory etiquette all undermine effective COVID-19 prevention and control in correctional facilities [8].

According to the WHO, prisons and other closed settings where people reside together proximately can serve as sources of infection, amplification, and transmission of infectious illnesses both within the correctional facilities and the wider outside community [9], making prisons sites of critical health concern. Moreover, overcrowding, inadequate sanitation, limited access to fresh air and physical activity, and other factors amplify the transmission of infectious diseases, further exacerbating the poor health conditions of the inmates. In Indonesia, Article 12 of 1995 and Government Regulation No. 58 of 1999 guarantee detainees the right to physical care and healthcare services, with rehabilitation serving as the primary objective of imprisonment. As members of society, incarcerated individuals are entitled to the same standard of healthcare as the general population. In addition to prisoner assimilation and integration programs, the Indonesian government implemented a range of control measures, including routine rapid antigen and reverse transcription polymerase chain reaction (RT-PCR) testing of inmates and prison staff, COVID-19 vaccination, postponement of new inmate admissions, replacement of in-person visits with video calls, conduct of court hearings via video conferencing, and relocation of inmates from correctional facilities operating at more than 300% capacity to less crowded facilities [7]. The Directorate General of Correctional Facilities, under the Ministry of Law and Human Rights, actively worked to prevent and control the spread of COVID-19 through implementation of stringent policies. The Directorate General outlined these measures in Instruction No. PAS-08.OT.02.02 Year 2020 titled ‘Prevention, Handling, Control, and Recovery of COVID-19 in Technical Implementation Units of Correctional Facilities’ [10].

Large COVID-19 outbreaks with high attack rates have been reported among incarcerated populations in many countries. For example, COVID-19 attack rates of 52% and 74% have been reported in two correctional facilities in the United States of America (USA) [11,12]. Staff members were also affected, although at lower rates than incarcerated individuals. In one outbreak, one-third of hospitalized cases succumbed, highlighting the potential severity of COVID-19 in correctional settings [11].

Indonesia has more than 500 prisons housing approximately 271,000 incarcerated individuals. Despite this substantial prison population, there is limited published literature on COVID-19 outbreaks in Indonesian correctional facilities, and virtually no studies have focused on female prisoners [13]. Existing international evidence suggests that incarcerated women were disproportionately affected by the COVID-19 pandemic, with pre-existing vulnerabilities compounded by new and heightened stressors related to reproductive health, caregiving, and parenting responsibilities [14,15]. However, there is currently little to no information on the impact of COVID-19 on female prisoners in Indonesia. Therefore, this study examined the occurrence of COVID-19 among prisoners in the Women’s State Prison in Jakarta.

## 2. Methods

This study involved a secondary analysis of survey data collected by Rahimullah et al. (2023) that focused broadly on the relationship between contact history and COVID-19 incidence among female inmates [16]. The original study employed a quantitative cross-sectional design. Primary data were collected from inmates at the Women’s State Prison of Jakarta using a structured questionnaire administered between May and June 2023, Monday to Friday, during prison working hours (08:00 to 16:00). The questionnaire comprised sections on participant demographics, contact history, occurrence of COVID-19 among prisoners, COVID-19 preventive behaviors, and the availability of infrastructure and facilities. Written informed consent was obtained from all participants prior to data collection.

The questionnaire comprised two scoring domains: (a) 10 questions assessing COVID-19 preventive behaviors, including compliance with hand hygiene and facemask use, willingness to receive COVID-19 vaccination, access to rapid antigen testing, and related practices; and (b) 10 questions assessing access to hygiene amenities and facilities, including the availability of handwashing facilities with running water, antiseptic solutions, disinfectants, tissues, wipes and other resources (Supplementary Material). The scoring instrument was developed by the Indonesian Directorate General of Corrections based on the provisions outlined in Instruction No. PAS-08.OT.02.02 Year 2020 on Prevention, Handling, Control, and Recovery of COVID-19 in Technical Implementation Units of Correctional Facilities [10]. Responses were recorded as binary outcomes (“yes” or “no”). Each affirmative response (“yes”) was assigned a score of one, resulting in a possible score range of minimum 0 to maximum 10 for each domain.

The study examined epidemiological factors related to COVID-19 among detainees, including contact history, COVID-19 preventive behaviors, availability of infrastructure and access to amenities. COVID-19 diagnoses were established by administering antigen-detection rapid diagnostic tests (Ag-RDTs) to individuals exhibiting clinical features consistent with COVID-19 those with consistent symptoms of COVID-19, in accordance with the WHO Emergency Use Listing (EUL) recommendations [17]. These Ag-RDTs were routinely used in correctional facilities across the country, including the study site. Bivariate associations were assessed using the chi-square test, while multivariate logistic regression analysis was performed to identify factors independently associated with COVID-19 among inmates. Multiple logistic regression analysis was performed using the backward stepwise selection approach. All independent variables were entered into the initial multivariable model without prior screening by chi-square analysis (e.g., *p* < 0.25), as they were considered to be of potential clinical and epidemiological relevance.

Variables were sequentially removed based on statistical significance, with those showing no significant association with the dependent variable (COVID-19 occurrence; *p* > 0.05) considered for elimination. At each step, changes in the adjusted odds ratio (OR_adj_) of the remaining variables were evaluated. Variables whose removal resulted in a change of more than 10% in the adjusted odds ratio (ΔOR_adj_) were retained in the model as potential confounders. In this study, no non-significant variable produced a ΔOR_adj_ exceeding 10%; therefore, all (non-significant) variables were excluded from the final model.

Potential interaction effects were evaluated among the independent variables retained in the final model. However, because only one independent variable remained after model selection, assessment of interaction was not applicable.

The sample size was estimated using the two-proportion hypothesis formula of the WHO/Lemeshow [18]. We assumed the proportion of COVID-19 with no contact history among inmates to be 0.108 and the proportion among those with a history of contact to be 0.339:

$$n=\frac{{\left\{Z_{1-\alpha /2}\sqrt{(2P\left(1-P\right)}+Z_{1-\beta }\sqrt{P1(1-P1)+P2(1-P2)}\right\}}^{2}}{{\left(P1-P2\right)}^{2}}$$

$$n=\frac{{\left\{1.96\sqrt{\left(2\left(0.224\right)\left(1-0.224\right)\right)}+1.28\sqrt{0.339(1-0.339)+0.108(1-0.108)}\right\}}^{2}}{{\left(0.339-0.108\right)}^{2}}$$

Information:

*n* = minimum sample size.
$Z_{1-\alpha /2}$ = *Z* score with 95% CI.
$Z_{1-\alpha /2}^{2}$ = *Z* score with power of test of 90%.
*P*1 = proportion of COVID-19 with contact history among inmates (0.339) [19].
*P*2 = proportion of COVID-19 with no contact history among inmates (0.108).
*P* = average proportion of COVID-19 in the general population (0.224) [19].

Based on the sample size calculation, a minimum of 67 respondents were required for the study. To account for potential attrition due to inmate transfers, releases, and institutional movement restrictions, the sample size was increased by approximately 60%, resulting in a target sample of 105 respondents to be recruited using proportionate stratified random sampling across three blocks of the prison.

The Ethical Committee for Health Research, Indonesian Association of Public Health Experts, has assessed and granted ethical approval for this study, reference No: 124/KEPK-IAKMI/VII/2020. All respondents voluntarily provided written informed consent prior to enrolment in the study, with the prison authorities witnessing the consent process.

## 3. Results

### 3.1. Bivariate Analysis

Of the 105 respondents recruited, 48 were from Block A, 39 from Block B, and the remaining 18 from Block C. Overall, 37 respondents (35.2%) had a diagnosis of COVID-19, including 10 from Block A, 18 from Block B, and nine from Block C, while the remaining 68 respondents (64.8%) did not have COVID-19. The highest attack rate of COVID-19 was observed in Block B (49%), followed by Block A (27%) and Block C (24%). A total of 61 respondents (58.1%) reported a history of contact with a COVID-19 case: 18 (29%) were from Block A, 28 (46%) from Block B, and 15 (25%) from Block C (Figure 1). Among those with a contact history, 34 respondents (55.7%) reported having COVID-19, whereas only three respondents (6.8%) without a contact history were infected. This indicates a significant association between a history of exposure to a COVID-19 case and COVID-19 infection (OR 17.21 [95% CI: 4.8–61.6], *p* < 0.01) (Table 1).

Regarding compliance with COVID-19 preventive practices, 78 participants (74.3%) scored 10 and were classified as “very good” compliers, while the remaining 27 participants (25.7%) scored below 10 and were classified as “good” compliers. The median compliance score was 10 (range 0 to 10), mean was 9.42. Among those categorized as “good” compliers, 12 (44.4%) contracted COVID-19 compared with 25 (32.1%) among “very good” compliers. However, there was no statistically significant association between compliance with preventive practices and COVID-19 infection (OR 1.7 [95% CI: 0.69–4.15], *p* = 0.35) (Table 1).

With respect to access to facilities and amenities, 46 participants (43.8%) scored above eight and were considered to have “good” access to facilities and amenities, whereas the other 59 participants (56.2%) scored eight or lower and were considered to have “moderate” access to facilities and amenities. The median access score was 8 (range 0 to 10), mean 8.52. Among participants with “moderate” access to facilities and amenities, 22 (37.3%) contracted COVID-19, whereas among participants with “good” access to facilities and amenities, 15 (32.6%) had infection; this signifies an absence of association between the availability of facilities and amenities and COVID-19 (OR 1.23 [95% CI: 0.54–2.76]) (Table 1).

### 3.2. Multivariate Analysis

Following the interaction and confounding tests, the final stage of the multivariate analysis involved developing the final regression model. The results indicated that COVID-19 preventive behaviors and the availability of infrastructure and facilities did not act as confounding variables in the relationship between contact history and infection. Therefore, these variables were not retained in the final model, and contact history remained the only significant predictor of COVID-19 occurrence:

COVID-19 Occurrence = 2.845 + 17.210 (Contact History) + e

The initial phase of performing logistic regression analysis utilizing the risk factor model involved examining the interaction between the primary independent variables, confounding factors, and the dependent variable. Variables were said to interact if they had a *p*-value < 0.05 (Table 2). Selection was done by removing non-significant interaction variables gradually and sequentially, starting with the interaction variable with the most significant *p*-value. After gradually removing these variables, the interaction test’s final results are as follows in Table 3.

During the confounding assessment, the variable “Access to facilities and amenities” was initially removed from the model. The resulting change in the ORs of the primary independent variables was less than 10%, indicating that this variable did not act as a confounder and could therefore be excluded from the model. Subsequently, “COVID-19 prevention behaviors” was removed and similarly produced a change in ORs of less than 10%, confirming its non-confounding role. Following completion of interaction testing and confounding assessment, the final multivariable model was established. The results demonstrated that neither “COVID-19 prevention behavior” nor “Access to facilities and amenities” met the criterion for confounding and, therefore, were not retained in the final model.

## 4. Discussion

The study revealed a high incidence of COVID-19 among female inmates, with 35% of women incarcerated in a central Indonesian prison becoming infected. These findings are consistent with reports of major COVID-19 outbreaks in correctional facilities worldwide, where overcrowding, limited opportunities for physical distancing, and frequent close-contact interactions facilitate rapid viral spread. For example, a large outbreak in a correctional facility in Utah, USA, was likely initiated by an asymptomatic healthcare provider and resulted in more than 1300 inmate infections and 11 deaths [11]. Similarly, a Delta variant outbreak in a federal prison in Texas infected 74% of inmates, with unvaccinated inmates experiencing higher rates of infection and severe disease, underscoring the importance of vaccination and layered prevention strategies, with higher infection and severe disease rates among unvaccinated individuals [12].

Our study also found a statistically significant association between prior contact with a confirmed COVID-19 case and subsequent COVID-19 infection among female prisoners in Indonesia (*p* < 0.01). Participants who reported exposure to an infected individual were 17.21 times more likely to contract COVID-19 than those without such exposure. This finding is consistent with previous studies demonstrating that close contact with an infected person is one of the strongest risk factors for SARS-CoV-2 infection [20]. Therefore, in addition to taking steps to prevent person-to-person exposure, contact tracing to contain an outbreak in the early phase of the outbreak is important [21]. An individual is considered to have been exposed to COVID-19 if they live, work with an infected person, have close contact for at least 15 min, or provide care without appropriate personal protective equipment (PPE) [22].

Preventive behaviors and the availability of infrastructure and facilities play a key role in mitigating COVID-19. To interrupt the transmission of COVID-19, the Centers for Disease Control and Prevention (CDC) recommend the consistent use of facemasks, regular hand hygiene, adherence to proper respiratory etiquette, and, whenever feasible, avoiding crowded gatherings and close contact with individuals who are ill. In localities experiencing community transmission, maintaining physical distance is also strongly advised. In addition, inadequate infrastructure and access to facilities can impede the adoption and maintenance of appropriate hygienic practices. Access to safe drinking and usable water, sanitation, and hygiene (WASH) services is crucial for preventing infectious diseases and protecting public health during epidemics, including the COVID-19 pandemic [23]. Individuals dwelling in inadequate housing conditions (such as prisons) often face challenges maintaining effective physical distancing and personal hygiene, which may aggravate their susceptibility to infection and contribute to more severe health outcomes associated with COVID-19 [24].

Interestingly, this study found no statistically significant association between preventive practices and COVID-19 infection among female prisoners (*p* = 0.35). These findings contrast with those reported by Payne et al. (2020), who observed lower infection rates among another institutional population (US Navy servicemen) who consistently adhered to preventive measures such as wearing face coverings, avoiding shared spaces, and maintaining physical distancing, compared with those who did not follow these practices [25]. The discrepancy may reflect differences in environmental conditions between community and correctional settings, highlighting the unique challenges of implementing and sustaining effective infection prevention measures in prisons. Together, these results suggest that while individual preventive behaviors remain important, they should be complemented by broader institutional interventions, such as reducing overcrowding, improving ventilation, strengthening infection control protocols, and ensuring timely identification and isolation of cases, to effectively limit COVID-19 transmission in correctional facilities.

However, the effectiveness of preventive measures, such as face mask use and hand hygiene, remains inconclusive in other crowded settings. A systematic review of 10 studies involving >5000 participants found that facemask use was not significantly associated with the risk of respiratory tract infections (RTIs) compared with no facemask use; however, the overall risk ratio (RR) favored wearing facemasks (RR = 0.78; 95% CI = 0.55–1.10) [26]. Also, a pilot randomized controlled trial that assessed hand hygiene against RRIs among 500 Umrah pilgrims found no significant difference in RTI rates between those given alcohol-based hand rub and controls [27]. A Cochrane review of 78 randomized trials found no clear evidence that medical or surgical masks reduce RTIs, including COVID-19; hand hygiene modestly reduced respiratory illness [28]. Despite the uncertainty of effectiveness, appropriate preventive practices remain essential for reducing SARS-CoV-2 transmission, particularly by limiting the spread of infectious respiratory droplets and aerosols generated during coughing, sneezing, talking, singing, or breathing, and compliance should be ensured [29]. It is noteworthy to mention that the symptoms of COVID-19 may differ based on an individual’s prior immunity, including vaccination status, or exposure to a novel variant [30], and virus transmission may occur regardless of whether the patients are asymptomatic, presymptomatic, or symptomatic [31].

Finally, no statistically significant association was observed between access to facilities and amenities and COVID-19 infection (*p* = 0.77). The absence of a significant relationship may be attributed to overall lower quality of the facilities in this setting, even though quantitatively, provision of facilities in the prison may have been adequate, as reflected by a decent mean facility score of 8.52 obtained from the questionnaire survey. In contrast, Ahmad et al. (2020) indicated that a 5% increase in the proportion of households experiencing poor housing conditions was associated with a 50% elevated risk of COVID-19 incidence and a 42% higher risk of mortality associated with COVID-19 in the USA [24]. Housing quality factors, including overcrowding, access to adequate sanitation and plumbing, and indoor air and water quality, may influence the effectiveness of COVID-19 prevention and control measures. Consequently, individuals dwelling in substandard housing conditions may face challenges in maintaining physical distancing and personal hygiene, thereby increasing their vulnerability to COVID-19.

This is a unique study to describe the occurrence of COVID-19 among female prisoners in Indonesia; however, a few key limitations should be acknowledged. The bureaucracy to access prisons to conduct research is complex in the Indonesian socio-politico-cultural system and ethically challenging. We could only collect selected data approved by the ethics committee, and several potentially important confounding variables were not collected; these include date of birth (or age), pre-existing medical conditions, vaccination status, duration of incarceration, prison occupancy, earlier outbreaks within specific prison blocks, and staff-related exposures could all influence COVID-19 occurrence. These limit the scope and generalizability of the study. Future endeavors are needed to minimize these shortcomings. Because this study employed a cross-sectional design, exposures and outcomes were measured simultaneously, limiting the ability to establish temporal sequencing and preventing conclusions regarding causality. Finally, this study relied on a generic instrument developed by the Indonesian Directorate General of Corrections that was not subjected to further validation or reliability testing. Consequently, the extent to which the instrument accurately captured the constructs of interest and produced consistent measurements remains uncertain, which may have influenced the study findings. Future endeavors are needed to minimize these shortcomings. Despite these limitations, the study offers notable novelty by generating previously unavailable evidence on COVID-19 infection and adherence to preventive behaviors among incarcerated women in Indonesia—a population for whom no prior empirical data exist. This contribution fills a critical gap in the literature and provides a foundation for future research and policy development.

Future studies should explore additional variables that were not assessed in this study, including population density, cough and sneeze etiquette, and environmental factors such as indoor air quality and ventilation. Expanding the study population to include prison personnel and incorporating qualitative methods such as direct interviews may also provide a more comprehensive understanding of COVID-19 transmission dynamics within correctional facilities in Indonesia. To further strengthen infection prevention and control efforts, the Women’s State Prison of Jakarta may consider implementing cohort-based outdoor activities to minimize interpersonal contact, improving the quality and frequency of cleaning and disinfection of high-touch surfaces, risk-based color coding of residential blocks (e.g., green, yellow, and red zones), promoting a healthy lifestyle among inmates through the promotion of clean and healthy living practices, and providing regular updates to prisoners and staff regarding COVID-19 cases within the facility.

## 5. Conclusions

This study found that more than one-third of the female prisoners in Indonesia reported having been diagnosed with COVID-19. Most participants (74%) demonstrated good compliance with COVID-19 preventive measures, while over 43% reported adequate availability of facilities and amenities to support prevention efforts.

Participants with a history of exposure to an infected individual were 17 times more likely to contract COVID-19 than those without such history. These findings have important policy implications for the prevention and control of infectious disease outbreaks in female correctional facilities in Indonesia. In particular, they highlight the critical importance of identifying, monitoring, and managing exposure to infected individuals as a key strategy for reducing disease transmission among incarcerated populations.

## Figures and Tables

**Figure 1:**
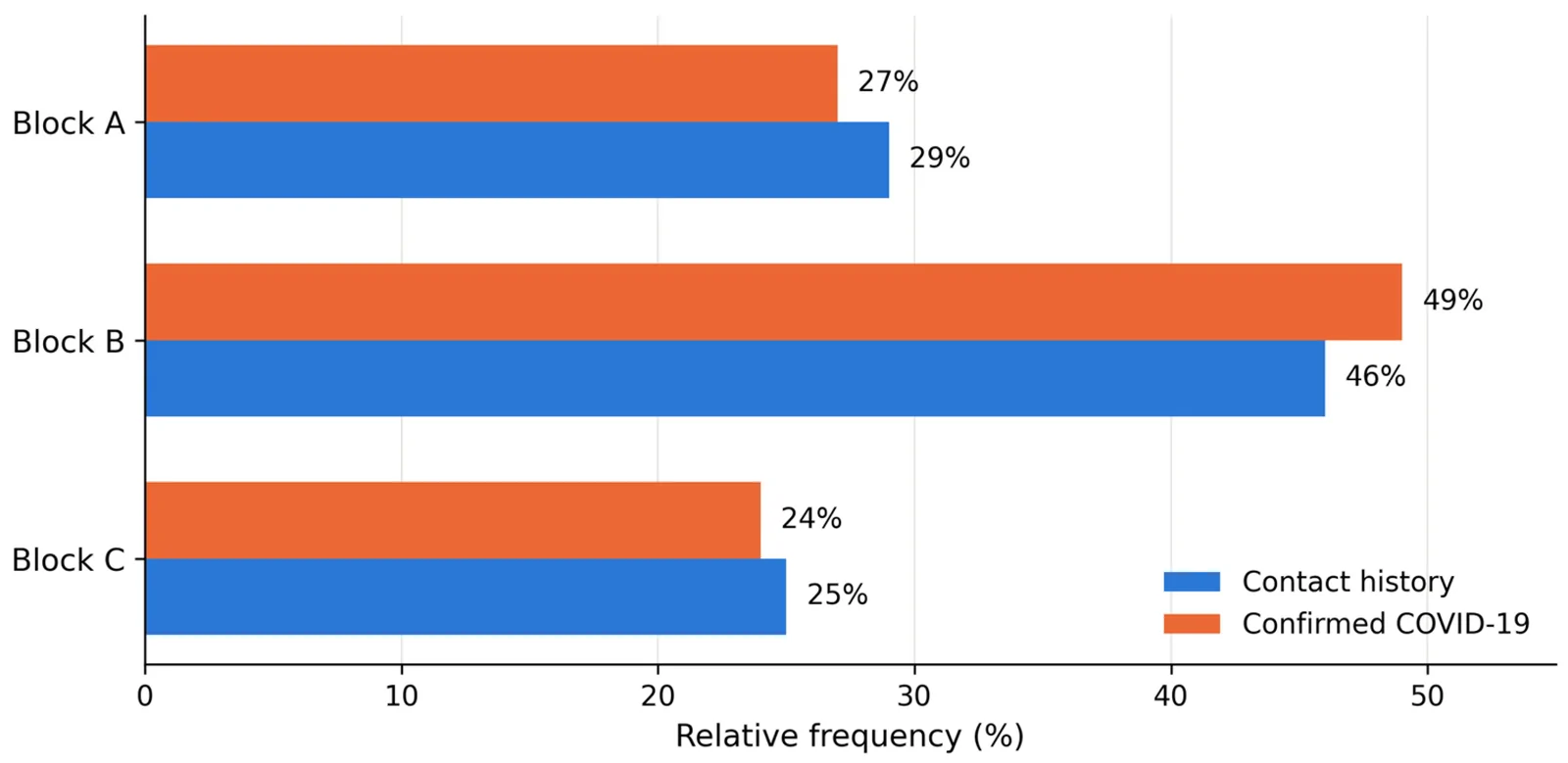
Distribution Chart of Contact History and COVID-19 Cases among Inmates of the Women’s State Prison of Jakarta based on Cell Blocks.

**Table 1:** Bivariate Analysis Results of COVID-19 Occurrence with Contact History, COVID-19 Prevention Behaviors, and Availability of Infrastructure and Facilities in the Women’s State Prison of Jakarta.

Variable	COVID-19 Occurrence				Total		Bivariate		Multivariate	
	Yes		No				OR_crude_ (95% CI)	*p*-Value	OR_adj_ (95% CI)	*p*-Value
	*n*	%	*n*	%	*n*	%				
**Contact History**							17.21 (4.8–61.6)	<0.01	17.21 (4.80–61.68)	<0.01
Yes	34	55.7	27	44.3	61	100				
No	3	6.8	41	93.2	44	100				
**COVID-19 Prevention Behaviors**							1.70 (0.69–4.15)	0.35	n/a	n/a
Good	12	44.4	15	55.6	27	100				
Very good	25	32.1	53	67.9	78	100				
**Access to facilities and amenities**							1.23 (0.54–2.76)	0.77	n/a	n/a
Moderate	22	37.3	37	62.7	59	100				
Good	15	32.6	31	67.4	46	100				

**Table 2:** Independent Variables for the Interaction Test.

Independent Variables	*p*-Value	OR
Contact History	<0.01	32.78
COVID-19 Prevention Behaviors	0.14	7.03
Availability of Infrastructure and Facilities	0.97	1.06
Contact History by COVID-19 Prevention Behaviors	0.27	0.20
Contact History by Availability of Infrastructure and Facilities	0.89	1.23

**Table 3:** Final Results of the Interaction Test.

Independent Variables	*p*-Value	OR
Contact History	< 0.01	18.15
COVID-19 Prevention Behaviors	0.23	1.93
Access to Facilities and Amenities	0.60	1.29

## Data Availability

The original contributions presented in this study are included in the article/Supplementary Material. Further inquiries can be directed to the corresponding author.

## Supplementary Materials

The following supporting information can be downloaded at: https://www.mdpi.com/article/10.3390/tropicalmed11100268/s1, File S1: The scoring instrument used in this study.
