# Supplementary material for: Addressing COVID-19 in Inmates at Women’s State Prison in Indonesia
Source: Trop Med Infect Dis. 2026 Sep 27;11(10):268. doi: 10.3390/tropicalmed11100268 (PMC13709363; doi:10.3390/tropicalmed11100268)
Supplement: Supplementary file 1 [file tropicalmed-11-00268-s001.zip › tropicalmed-4411209-supplementary.pdf]

File S1: The scoring instrument used in this study

No.	Independent Variables' Questions	Best Answer (Score=1)
	**Contact History**	**(Max Score=8)**
1.	Have you been exposed to a person with confirmed COVID-19 infection within the last 14 days?	Yes/No
2.	Has your cellmate ever tested positive for COVID-19?	Yes/No
	If yes, please specify the date of diagnosis (Date: DD/MM/YYYY)	
3.	Have you been in the same room as an inmate with confirmed COVID-19 at a distance of 1 to 2 meters for more than 15 minutes?	Yes/No
4.	Have you been in the same room as a prison officer with confirmed COVID-19 at a distance of 1 to 2 meters for more than 15 minutes?	Yes/No
5.	Do you have a history of face-to-face or close contact with a probable or confirmed COVID-19 case among inmates?	Yes/No
6.	Have you had face-to-face or other close contact with an inmate who was a probable or confirmed case of COVID-19?	Yes/No
7.	Do you have a history of direct physical contact with a probable or confirmed COVID-19 case among inmates?	Yes/No
8.	Do you have a history of direct physical contact with a probable or confirmed COVID-19 case among prison officers?	Yes/No
	**COVID-19 Prevention Behavior**	**Very Good (Max score=10)**
1.	Have you changed your daily habits during the COVID-19 pandemic to reduce your risk of infection?	Yes/No
2.	Do you wear a facemask when you are examined by a a healthcare professional?	Yes/No
3.	Do you wear a facemask when you are outside your cell?	Yes/No

No.	Independent Variables' Questions	Best Answer (Score=1)
4.	Do you wash your hands or use hand sanitizer after being examined by a healthcare professional?	Yes/No
5.	Do you wash your hands or use hand sanitizer when you are outside your cell?	Yes/No
6.	Do you wash your hands or use hand sanitizer after toilet action?	Yes/No
7.	Do you wash your hands or use hand sanitizer before meal?	Yes/No
8.	Are you willing to get vaccinated against COVID-19?	Yes/No
9.	Have you sought information and safety guidelines about COVID-19?	Yes/No
10.	Have you ever been tested for COVID-19 (e.g., using a rapid antigen test)?	Yes/No
	**Access to Facilities and Amenities**	**Very Sufficient (Max score=10)**
1.	Do you have access to a handwashing facility with running water?	Yes/No
2.	Is hand soap available?	Yes/No
3.	Is hand sanitiser available?	Yes/No
4.	Are dry tissues available?	Yes/No
5	Are wet tissues available?	Yes/No
6.	Are antiseptic products available?	Yes/No
7.	Are disinfectants available?	Yes/No
8.	Is your cell kept clean?	Yes/No
9.	Is adequate ventilation available?	Yes/No

No.	Independent Variables' Questions	Best Answer (Score=1)
10.	Is natural lighting (sunlight) available?	Yes/No
